# Overexpression of O‐polysaccharide chain length regulators in Gram‐negative bacteria using the Wzx‐/Wzy‐dependent pathway enhances production of defined modal length O‐polysaccharide polymers for use as haptens in glycoconjugate vaccines

**DOI:** 10.1111/jam.13772

**Published:** 2018-05-15

**Authors:** N. Hegerle, J. Bose, G. Ramachandran, J.E. Galen, M.M. Levine, R. Simon, S.M. Tennant

**Affiliations:** ^1^ Center for Vaccine Development and Institute for Global Health University of Maryland School of Medicine Baltimore MD USA; ^2^ Department of Medicine University of Maryland School of Medicine Baltimore MD USA; ^3^ Department of Pediatrics University of Maryland School of Medicine Baltimore MD USA

**Keywords:** glycoconjugate, haptens, LPS (Lipopolysaccharide), O‐polysaccharide, *pseudomonas*, *salmonella*, vaccines

## Abstract

**Aims:**

O‐polysaccharide (OPS) molecules are protective antigens for several bacterial pathogens, and have broad utility as components of glycoconjugate vaccines. Variability in the OPS chain length is one obstacle towards further development of these vaccines. Introduction of sizing steps during purification of OPS molecules of suboptimal or of mixed lengths introduces additional costs and complexity while decreasing the final yield. The overall goal of this study was to demonstrate the utility of engineering Gram‐negative bacteria to produce homogenous O‐polysaccharide populations that can be used as the basis of carbohydrate vaccines by overexpressing O‐polysaccharide chain length regulators of the Wzx‐/Wzy‐dependent pathway.

**Method and Results:**

The O‐polysaccharide chain length regulators *wzzB* and *fepE* from *Salmonella* Typhimurium I77 and *wzz2* from *Pseudomonas aeruginosa* PAO1 were cloned and expressed in the homologous organism or in other Gram‐negative bacteria. Overexpression of these Wzz proteins in the homologous organism significantly increased the proportion of long or very long chain O‐polysaccharides. The same observation was made when *wzzB* was overexpressed in *Salmonella* Paratyphi A and *Shigella flexneri,* and *wzz2* was overexpressed in two other strains of *P. aeruginosa*.

**Conclusions:**

Overexpression of Wzz proteins in Gram‐negative bacteria using the Wzx/Wzy‐dependant pathway for lipopolysaccharide synthesis provides a genetic method to increase the production of an O‐polysaccharide population of a defined size.

**Significance and Impact of the Study:**

The methods presented herein represent a cost‐effective and improved strategy for isolating preferred OPS vaccine haptens, and could facilitate the further use of O‐polysaccharides in glycoconjugate vaccine development.

## Introduction

Lipopolysaccharides (LPS) are the main constituents of the Gram‐negative bacterial (GNB) outer membrane. The basic structure of LPS is comprised of three distinct units. The lipid A serves as a membrane anchor and possesses endotoxic properties. The core polysaccharide is covalently attached to lipid A and is mostly conserved within individual bacterial species. The final component is the O‐polysaccharide (OPS) that extends from the core and is a variable polymer of repeating saccharide units for which the chemical structure defines the associated bacterial serogroup (Erridge *et al*. 2002). While most GNB (e.g. *Salmonella enterica*,* Shigella flexneri*,* Pseudomonas aeruginosa*,* Klebsiella pneumoniae*) possess OPS, some GNB (e.g. *Bordetella pertussis*,* Neisseria meningitidis*,* Haemophilus influenzae*) produce lipo‐oligosaccharide or LOS, that is short and characterized by only a few saccharide repeats.

Unlike the conserved core region, the OPS can be highly variable between bacteria of the same species and infection with a GNB pathogen can induce production of highly specific anti‐OPS antibodies that recognize the abundant surface‐associated OPS of GNB human pathogens (e.g. *K. pneumoniae* (Podschun and Ullmann 1998), *S. enterica* (Wattiau *et al*. 2011), *P. aeruginosa* (Al‐Dujaili and Harris 1974)). Antibodies specific for OPS have shown promise as vaccine antigens for several bacterial pathogens, whereby they have protected against challenge with the homologous pathogen in preclinical animal models (Svenson and Lindberg 1981; Watson *et al*. 1992; Simon *et al*. 2011). Glycoconjugate vaccines of *S*. Paratyphi A OPS with tetanus toxoid (TT) elicited bactericidal antibodies in human trials in Vietnam (Konadu *et al*. 2000). A *Shigella sonnei* core and OPS (COPS) glycoconjugate with recombinant *P. aeruginosa* exoprotein A demonstrated efficacy against clinical disease due to the homologous OPS expressing pathogen in a double‐blind vaccine‐controlled randomized clinical trial for efficacy conducted among Israeli army recruits (Cohen *et al*. 1997). However, despite these promising studies there is currently no licensed OPS‐based human vaccine.

As isolated antigens, polysaccharides are generally poorly immunogenic and usually considered T‐independent antigens that fail to activate maturation of memory B cells and antibody class switching (Finco and Rappuoli 2014). Chemical linkage, or conjugation, of the OPS to a carrier protein has been shown to improve its immunogenicity (Campbell *et al*. 1996; Simon *et al*. 2011) by engagement of carrier protein‐specific T cells, and enabling the presentation of the OPS‐peptide on MHCII for engagement of polysaccharide‐specific CD4^+^ T‐helper cells (Avci *et al*. 2011). Variability in O polymer size expressed by the bacteria used as the source of OPS antigen represents a practical challenge, however, for vaccine development. The size of the polysaccharide hapten can play an important role in the immune response to carbohydrate vaccines (Kubler‐Kielb *et al*. 2010; Zhang *et al*. 2013; Broker *et al*. 2016). Individual bacterial strains may also express a wide range of OPS sizes that, in some instances, may not include the desired target size for vaccine development.

Two major pathways have been described for the synthesis and export of GNB polysaccharides: the Wzx‐/Wzy‐dependant pathway (Islam and Lam 2014) (e.g. *S. enterica*,* S. flexneri* and *P. aeruginosa*) and the ABC transporter‐dependent pathway (e.g. *Klebsiella pneumoniae* and some *Escherichia coli*) (Greenfield and Whitfield 2012). For OPSs synthesized through the Wzx‐/Wzy‐dependent pathway, the Wzz family proteins are membrane‐associated O antigen chain length regulators expressed in the periplasm of GNBs that control the modal number of OPS repeats polymerized by the Wzy protein. Some species encode several different Wzz proteins, each producing a unique OPS modal length (Daniels *et al*. 2002; Murray *et al*. 2003). In *S*. Typhimurium, WzzB controls the biosynthesis of long‐chain OPS with average chain lengths of 16–35 repeat units of polysaccharide monomer while FepE controls the biosynthesis of very long OPS with average chain lengths of >100 repeat units (Murray *et al*. 2003). In *P. aeruginosa*, Wzz1 controls the synthesis of two distinct OPS population with modal OPS lengths of 12–16 and 22–30 repeat units while Wzz2 is responsible for the biosynthesis of long‐chain O‐polysaccharide with average chain lengths of 40–50 repeat units of polysaccharide monomer (Daniels *et al*. 2002). The endogenous expression of these OPS biosynthesis proteins can be subject to control by growth phase and environmental conditions (Carter *et al*. 2007; Bravo *et al*. 2008).

Murray *et al*. demonstrated that, in *S*. Typhimurium, complemention of *wzz* mutants with homologous plasmid‐encoded *wzz* genes leads to the synthesis of OPS incorporating the number of repeat units governed by the heterologous expressed Wzz protein (Murray *et al*. 2003). The overexpression of selected *wzz* genes in wild‐type strains should thus enable a bias for OPS size towards a population mainly comprising polysaccharides of a modal length defined by the overexpressed Wzz. In this article, we report the cloning of Wzz proteins from *S*. Typhimurium and *P. aeruginosa* and describe the impact of Wzz overexpression on the size distribution of the OPS population in homologous or heterologous strains using the Wzx‐/Wzy‐dependent pathway for OPS synthesis.

## Materials and methods

### Bacterial strains, medium, and growth

The strains used in this study are described in Table 1. All strains were maintained on HS bacteriological media (5 g l^−1^ sodium chloride, 10 g l^−1^ soytone [Teknova, Hollister, CA, USA], 5 g l^−1^ Hy‐yest [Kerry, Beloit, WI, USA]) at 37°C. Growth medium for all *guaBA* mutants was supplemented with guanine (0·001% w/v). Kanamycin or carbenicillin was added at a final concentration of 50 *μ*g ml^−1^ for pSEC10 and pSE280 maintenance, respectively, in *Salmonella* spp. and *Shigella* spp. Carbenicillin was added at a final concentration of 250 *μ*g ml^−1^ for pUCP19 maintenance in *P. aeruginosa* strains PAO1, IATS O6 and IATS O11. Isopropyl *β*‐D‐1‐thiogalactopyranoside (IPTG; Teknova, Hollister, CA, USA) was added at 0·1 mol l^−1^ final concentration for induction of *wzz2* overexpression in *P. aeruginosa* strains.

**Table 1:** Bacterial strains used in this study

Bacterial strains name	Mutations	Characteristics	References
*Salmonella* Paratyphi A CVD 1902	Δ*guaBA* Δ*clpX*	Candidate live attenuated vaccine, serogroup A (O:2)	ClinicalTrials.gov NCT01129453
*Salmonella* Typhimurium CVD 1925	Δ*guaBA* Δ*clpP* Δ*fliD* Δ*fljB*	Reagent strain for OPS purification for conjugate vaccine, serogroup B (O:4)	(Tennant *et al*. 2011)
*Salmonella flexneri* CVD 1208S	Δ*guaBA* Δ*set* Δ*sen*	Candidate live attenuated vaccine, serotype 2a	(Kotloff *et al*. 2007)
*Pseudomonas aeruginosa* PAO1	None	O5	(Holloway 1969)
*Pseudomonas aeruginosa* IATS O6	None	O6	(Lam *et al*. 1987)
*Pseudomonas aeruginosa* IATS O10	None	O10	(Lam *et al*. 1987)

### DNA techniques

#### DNA amplification

The primers used in this study are listed in Table 2. Amplification of *wzzB* and *fepE* from *S*. Typhimurium I77 and *wzz2* from *P. aeruginosa* PAO1 was conducted in 50 *μ*l final volume using Vent^®^ DNA polymerase (New England Biolabs, Ipswich, MA, USA) according to the manufacturer's protocol. PCR products were purified using a QIAQUICK PCR Purification Kit (Qiagen, Germantown, MD, USA) according to the manufacturer's protocol.

**Table 2:** Primers used in this study

Primer name	Target	Strain	Primer sequence (5′‐>3′)^a^
*wzzB*_F‐BamHI	*wzzB*	I77	AAA**GGATCC**ATGACAGTGGATAGTTATACG
*wzzB*_R‐Pst	*wzzB*	I77	AAA**CTGCAG**TTACAAGGCTTTTGGCTTATAG
*fepE*_F‐BamHI	*fepE*	I77	ATA**GGATCC**ATGCCATCTCTTAATGTAAAACAAGA
*fepE*_R‐NheI	*fepE*	I77	ACA**GCTAGC**TCAGACTAACCGTTCATCTATCGC
*wzz2*_F‐BamHI	*wzz2*	PAO1	ATCAAT**GGATCC**TATGCCTTCCTCACAGCTTCC
*wzz2*_R‐EcoRI	*wzz2*	PAO1	AATGCT**GAATTC**TCAGGTCCCTGAAAGGCTC

a The bold letters in the primer sequence represent the restriction sites.

#### Plasmid DNA preparation

Plasmids were purified using the Wizard^®^ Plus SV Minipreps DNA purification kit (Promega, Madison, WI, USA).

#### DNA digestion and ligation

Purified PCR products and plasmids were digested with the appropriate restriction endonucleases (New England Biolabs, Ipswich, MA, USA) and ligated with T4 DNA‐ligase (New‐England Biolabs, Ipswich, MA, USA). All plasmids were verified by sequencing (Genewiz, South plainfield, NJ, USA) for the correct insertion of *wzzB, fepE* and *wzz2*.

### Genetic engineering of strains that overexpress wzzB and fepE

#### Expression of wzzB from a low copy number plasmid

*wzzB* from *S*. Typhimurium I77 was first cloned into the high copy number, ampicillin‐resistant plasmid pSE280. The 1‐kbp PCR product was digested with BamHI and PstI, ligated to the similarly digested pSE280 (3·9 kbp) and electroporated into *E. coli* DH5alpha. *wzzB* from pSE280‐*wzzB* was then cloned into pSEC10 (7·2 kbp), a low copy number expression plasmid which encodes resistance to kanamycin (Stokes *et al*. 2007). A 984‐bp fragment encoding *wzzB* was obtained by digesting pSE280‐*wzzB* with BamHI and NheI and was ligated to the similarly digested pSEC10. The resultant pSEC10‐*wzzB* plasmid was transformed into *S*. Paratyphi A CVD 1902, *S*. Typhimurium CVD 1925 and *S. flexneri* CVD 1208S.

#### Expression of fepE from pSEC10

*fepE* amplified from *S*. Typhimurium I77 was digested with BamHI and NheI and inserted as a 1·1‐kbp fragment into the similarly digested pSEC10 plasmid. The 7408‐bp pSEC10‐*fepE* plasmid was subsequently transformed into *S*. Paratyphi A CVD 1902.

#### Expression of wzz2 from pUCP19

*wzz2* was amplified from *P. aeruginosa* PAO1 and was cloned into the ampicillin‐resistant low copy number expression shuttle vector pUCP19 (4·6 kbp) (Schweizer 1991). The BamHI and EcoRI‐digested 1·3‐kbp PCR product was ligated to the similarly digested pUCP19 vector and the 5872‐bp pUCP19‐*wzz2* plasmid was transformed into *P. aeruginosa* PAO1, IATS O6 and IATS O10.

### LPS isolation and visualization

Overnight bacterial cultures of *S*. Typhimurium CVD 1925, *S*. Paratyphi A CVD 1902 and *S. flexneri* CVD 1208S were adjusted to an OD_600 nm_ of 1 and then 2 ml of culture was centrifuged at 15 000 *× **g*** for 2 min at 4°C. The supernatant was removed and the bacterial pellet was resuspended in 100 *μ*l lysis buffer (0·1 M Tris‐HCl, pH 6·8, 2% SDS, 10% glycerol, 4% 2‐mercapthoethanol). The sample was boiled at 100°C for 10 min to lyse the cells. Proteins were digested by adding 25 *μ*g Proteinase K and incubating the sample for one h at 60°C. The sample was boiled for 10 min and then allowed to cool on ice. Twenty microlitres of the sample was electrophoresed on a 4%–15% Mini Protean TGX stain‐free gel (BioRad Laboratories, Hercules, CA, USA) with the CandyCane Glycoprotein ladder (Life Technologies, Carlsbad, CA, USA). LPS was isolated from *P. aeruginosa* strains as described (Hitchcock and Brown 1983) and electrophoresed on a 12% NuPAGE^®^ Bis‐Tris acrylamide gel (Thermo Fisher Scientific, Waltham, MA, USA). LPS was stained using Pro‐Q Emerald 300 LPS Gel Stain (Thermo Fisher Scientific, Waltham, MA, USA) per the manufacturer's instructions and visualized with a ChemiDoc™ MP instrument using Image Lab 5·1 software (BioRad Laboratories, Hercules, CA, USA). We categorized the OPS as short, medium, long or very long OPS based on their apparent electrophoretic mobility in the acrylamide gel.

### Densitometry and statistical analysis

Images of stained LPS gels were analysed with the built in densitometry analysis tool of Image Lab 5·1 (BioRad Laboratories, Hercules, CA). The results are presented as the mean ± standard error of the mean (SEM) of three independent experiments. Two‐way or one‐way anova was performed using GraphPad Prism ver. 6·00 for Windows (GraphPad Software, La Jolla, CA, USA). A difference was considered statistically significant when *P* ≤ 0·05 and is represented by three asterisks (***) on the figures.

## Results

### Plasmid construction

**Figure 1:**
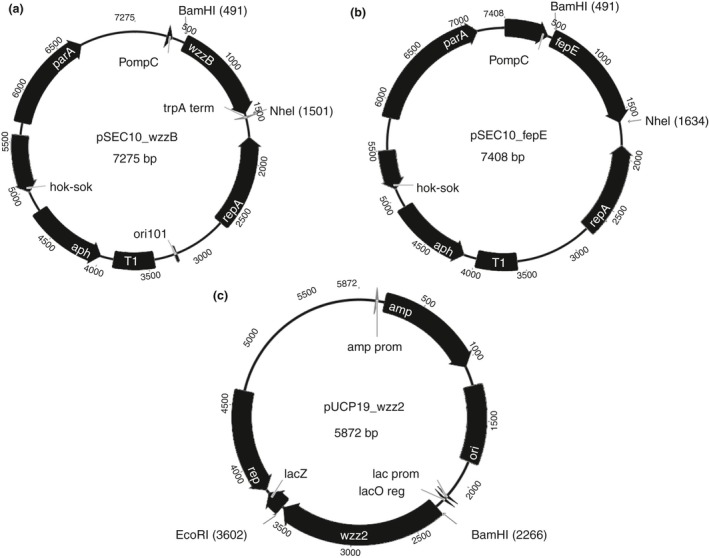
Schematic representation of (a) pSEC10‐*wzzB* (b) pSEC10‐*fepE* and (c) pUCP19‐*wzz2*.

*Salmonella* Typhimurium *wzzB* and *fepE* were cloned into pSEC10 downstream of the *ompC* promoter and confirmed by sequencing (Fig. 1a,b). *P. aeruginosa wzz2* was cloned into the pUCP19 plasmid downstream of the *lac* promoter and confirmed by sequencing (Fig. 1c). *wzz2* expression was induced by the addition of 0·1 mol l^−1^ IPTG to the growth medium.

### Overexpression of *wzzB* and *fepE* in the homologous organism

**Figure 2:**
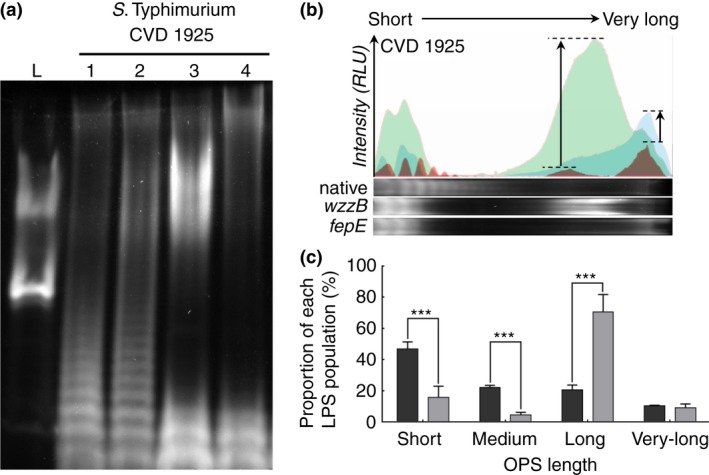
(a) Visualization of LPS produced by *Salmonella* Typhimurium CVD 1925 (lane 1), carrying the empty pSEC10 plasmid (lane 2), carrying pSEC10‐*wzzB* (lane 3) and carrying pSEC10‐*fepE* (lane 4). (b) Distribution of the signal intensity in lane 1 (*S*. Typhimurium CVD 1925, red), lane 3 (*S*. Typhimurium CVD 1925 [pSEC10‐*wzzB*]*,* green) and lane 4 (*S*. Typhimurium CVD 1925 [pSEC10‐*fepE*], blue). (c) Proportion of each OPS population relative to the total signal intensity per lane for *S*. Typhimurium CVD 1925 (dark grey bars) and *S*. Typhimurium CVD 1925 (pSEC10‐*wzzB)* (light grey bars). [Colour figure can be viewed at wileyonlinelibrary.com]

Overexpression of *S*. Typhimurium *wzzB* from pSEC10‐*wzzB* in *S*. Typhimurium CVD 1925 resulted in a significant increase in the proportion of long OPS and concomitant decrease in the proportion of short and medium length OPS (Fig. 2) compared to *S*. Typhimurium CVD 1925. Similar results were observed when *wzzB* was overexpressed from pSE280 or with addition of sodium chloride to the culture medium (data not shown), suggesting that overexpression of *wzzB* from pSEC10 in noninduced growth conditions is already saturating. Similarly, the overexpression of *fepE* resulted in an increase in the proportion of very long chain OPS (Fig. 2a,b). The empty plasmid did not have any impact on the OPS chain length (Fig. 2a).

### Expression of *S*. Typhimurium *wzzB* in *S*. Paratyphi A CVD 1902 and *S. flexneri 2a* CVD 1208S

**Figure 3:**
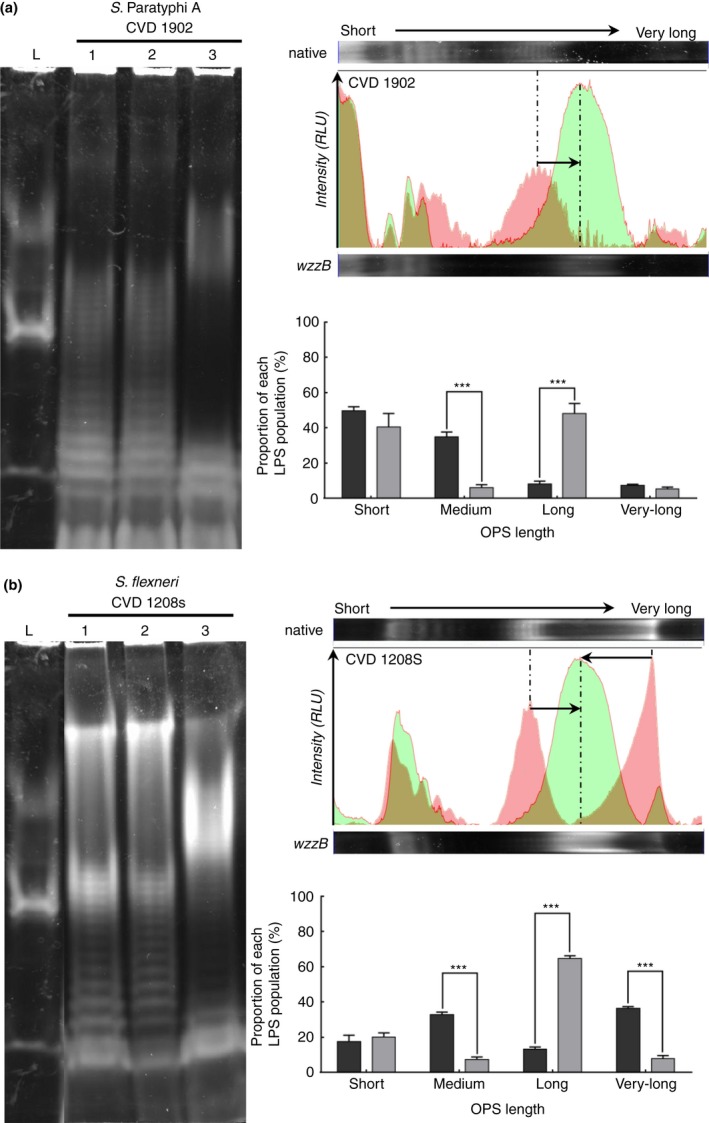
(a) Visualization of LPS produced by *Salmonella* Paratyphi A CVD 1902 (lane 1), *S*. Paratyphi A CVD 1902 (pSEC10) (lane 2) and *S*. Paratyphi A CVD 1902 (pSEC10‐*wzzB*) (lane 3) and visualization of the signal intensity distribution and proportion of each OPS population in *S*. Paratyphi A CVD 1902 (red histogram, dark grey bars) and *S*. Paratyphi A CVD 1902 (pSEC10‐*wzzB*) (green histogram, light grey bars). (b) Visualization of LPS produced by *S. flexneri* CVD 1208S (lane 1), *S. flexneri* CVD 1208S (pSEC10) (lane 2) and *S. flexneri* CVD 1208S (pSEC10‐*wzzB*) (lane 3) and visualization of the signal intensity distribution and proportion of each OPS population in *S. flexneri* CVD 1208S (red histogram, dark grey bars) and *S. flexneri* CVD 1208S (pSEC10‐*wzzB*) (green histogram, light grey bars). [Colour figure can be viewed at wileyonlinelibrary.com]

In order to test whether overexpression of *S*. Typhimurium *wzzB* could confer similar modal length changes among other bacteria that use the Wzx‐/Wzy‐dependent LPS synthesis pathway, pSEC10‐*wzzB* was transformed into *S*. Paratyphi A CVD 1902, and into *S. flexneri* CVD 1208S. The overexpression of *S*. Typhimurium *wzzB* in these two strains led to the production of OPS with a modal chain length distribution similar to that observed for *S*. Typhimurium CVD 1925 (pSEC10‐*wzzB)* (Fig. 3). The OPS produced by *S*. Paratyphi A CVD 1902 (pSEC10‐*wzzB)* shifted to a longer modal length (Fig. 3a) while for *S. flexneri* CVD 1208S (pSEC10‐*wzzB*) a shift from short to very long OPS to a uniform high molecular weight species intermediate between the short and very long O chain length was observed (Fig. 3b). The empty plasmid did not induce any change in the LPS population compared to the parental strains (Fig. 3).

### Densitometry analysis of the OPS produced in strains overexpressing *S*. Typhimurium *wzzB*

**Figure 4:**
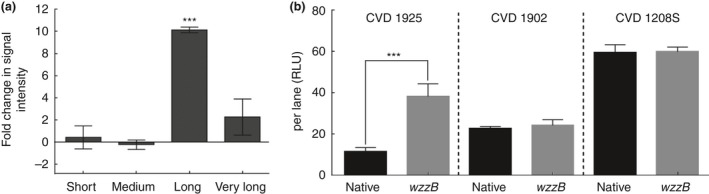
(a) Fold‐change in the signal intensity measured for each OPS population in *Salmonella* Typhimurium CVD 1925 (pSEC10‐*wzzB)* as compared to *S*. Typhimurium CVD 1925. (b) Total signal intensity measured by densitometry analysis in parental strains *S*. Typhimurium CVD 1925, *S*. Paratyphi A CVD 1902 and *Salmonella flexneri* CVD 1208S and in strains overexpressing *wzzB* from pSEC10.

Overexpression of *wzzB* induced a *c*. 10‐fold increase in the proportion of synthesized long‐chain OPS in *S*. Typhimurium CVD 1925 (Fig. 4). While the total amount of OPS produced remained equal between *S*. Paratyphi A CVD 1902 (pSEC10‐*wzzB*) and CVD 1208S (pSEC10‐*wzzB*) and their respective parental strains *S*. Paratyphi A CVD 1902 and *S. flexneri* CVD 1208S, *S*. Typhimurium CVD 1925 (pSEC10‐*wzzB)* had a significantly higher signal intensity than *S*. Typhimurium CVD 1925 (Fig. 4). Since all samples were normalized, the data suggest that *S*. Typhimurium CVD 1925 (pSEC10‐*wzzB)* actually synthesizes more OPS than its parental strain.

### Overexpression of *wzz2* in *P. aeruginosa* PAO1 and in heterologous strains of the same species, *P. aeruginosa* IATS O6 and IATS O10

**Figure 5:**
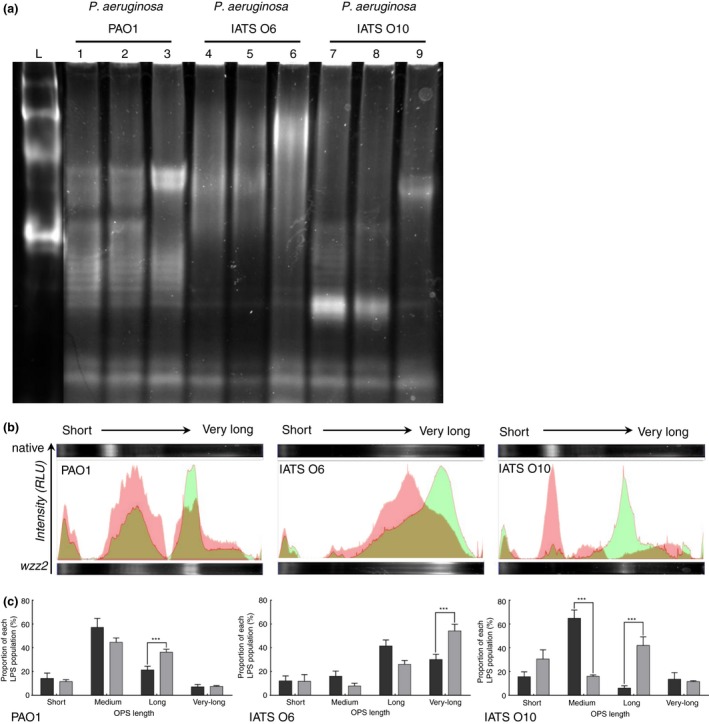
(a) Visualization of LPS produced by wild‐type *Pseudomonas aeruginosa* strains PAO1 (lane 1), IATS O6 (lane 4) and IATS O10 (lane 7), and the same strains carrying pUCP19 (lanes 2, 5 and 8 respectively) or pUCP19‐*wzz2* (lanes 3, 6 and 9 respectively). (b) Visualization of the signal intensity distribution for the wild‐type (red) and the *wzz2* overexpressing (green) PAO1, IATS O6 and IATS O10 strains. (c) Proportion of each OPS population relative to the total signal intensity per lane for wild‐type (dark grey) and *wzz2* overexpressing (light grey) strains of PAO1 (left), IATS O6 (middle) and IATS O10 (right). [Colour figure can be viewed at wileyonlinelibrary.com]

We applied the same strategy to control the OPS length in *P. aeruginosa*, another GNB that uses the Wzx‐/Wzy‐dependant pathway for LPS synthesis. Similar to the results found for *S*. Typhimurium CVD 1925, overexpression of *P. aeruginosa wzz2* in *P. aeruginosa* strain PAO1 caused a significant increase in the proportion of long‐chain OPS (Fig. 5). When *wzz2* was overexpressed in *P. aeruginosa* strain IATS O10 a significant increase in long‐chain LPS was balanced by a significant decrease in medium chain LPS (Fig. 5c) The overexpression of *wzz2* in *P. aeruginosa* IATS O6 lead to a significant increase in the proportion of very long chain OPS (Fig. 5).

## Discussion

As biological products, vaccines have moved steadily away from poorly defined and difficult to standardize preparations (e.g. heat or chemically inactivated whole‐cell vaccines), towards well‐defined products (e.g. subunit vaccines). Based on encouraging preclinical results, there is broad enthusiasm and intensive research efforts towards development of OPS‐based vaccines for a multitude of GNB pathogens (Svenson and Lindberg 1981; Watson *et al*. 1992; Cohen *et al*. 1997; Simon *et al*. 2011). Immunogenic and effective protein‐polysaccharide glycoconjugate vaccines are among the most complex vaccines to design due to several contributing factors including variability in the saccharide hapten size and carrier protein, linkage site between the saccharide and protein carrier, and chemistries required to link the hapten and carrier protein (Bardotti *et al*. 2008; Stefanetti *et al*. 2014; Broker *et al*. 2016). Control over the polysaccharide hapten size is thus important towards the development of glycoconjugate vaccines. Production of well‐defined and uniform lower molecular weight polysaccharide fragments for *Neisseria meningitidis* and *Haemophilus influenzae* type b (Hib) capsular polysaccharides has been accomplished by chemical or mechanical polysaccharide depolymerization, respectively, followed by size fractionation (Bardotti *et al*. 2008; Broker *et al*. 2016). Biochemical size fractionation has also been used for purified *Shigella* COPS, whereby a preferred immunogenic saccharide size population was identified (Robbins *et al*. 2009; Kubler‐Kielb *et al*. 2010; Zhang *et al*. 2013). *S*. Typhimurium OPS haptens of reduced size produced by enzymatic cleavage of S. Typhimurium COPS with a phage‐associated endorhamnosidase were used as glycoconjugate haptens to establish the minimal immunogenic repeat number (Svenson and Lindberg 1981). Chemical synthesis approaches have also been utilized to produce bacterial polysaccharide haptens of uniform size and composition (Aguilar‐Betancourt *et al*. 2008; Phalipon *et al*. 2009; Polonskaya *et al*. 2017). Manipulation of the OPS synthesis pathway represents an efficient and economical approach to achieve high yields of homogenous polysaccharides of a predetermined size, with clear advantages over the prior approaches.

The *S*. Typhimurium strain CVD 1925 produces predominantly short to medium molecular weight LPS with a characteristic laddering pattern under normal growth conditions (Fig. 2). Overexpression of the O antigen modal length regulator *wzzB* from a plasmid in this strain overcame this deficiency, resulting in the production of *c*. 10‐fold higher levels of long‐chain saccharide size. Overexpression of *fepE*, the very long O antigen modal length regulator, in this same strain also shifted the OPS population from predominantly short to very long. *S*. Paratyphi A, a serogroup A *Salmonella* that produces OPS that is structurally very similar to *S*. Typhimurium (Lindberg 1984), expresses almost entirely short‐chain OPS (Boyd *et al*. 2014). We found that expression of *wzzB* from *S*. Typhimurium CVD 1925 in *S*. Paratyphi A CVD 1902 shifted the saccharide population to long length LPS with the same molecular weight as that produced by *S*. Typhimurium CVD 1925. *S. flexneri* 2a expresses two separate chromosomally‐ and plasmid‐encoded *wzz* genes (e.g. *wzzB* and *wzz*_pHS‐2_ respectively) that specify either short or very long LPS types (Carter *et al*. 2009). High‐level expression of *S*. Typhimurium CVD 1925 *wzzB* by *S*. *flexneri* CVD 1208S supplanted almost fully the action of the endogenously encoded *Shigella* Wzz proteins, producing a homogenous population of intermediate molecular weight LPS. It has been suggested that the higher expression level of plasmid‐encoded OPS chain length regulators shifts the stoichiometry of the reaction and forces the system to use the recombinant Wzz protein instead of native Wzz proteins (Islam and Lam 2014). This might result in a competition between native Wzz proteins and WzzB for the engagement of Wzy and control of the O‐polysaccharide length. This finding provides a precedent for shifting the saccharide size from both long and short towards an intermediate and uniform size. When this strategy was applied to *P. aeruginosa* we found comparable results whereby the overexpression of PAO1 *wzz2* induced an increase in the modal length of the OPS populations in the three distinct strains. The overexpression of *wzz2* in *P. aeruginosa* IATS O6, however, produced an O antigen population with a higher molecular weight observed by SDS‐PAGE than that produced by *P. aeruginosa* PAO1 overexpressing *wzz2*. This discrepancy may be accounted for by differences in OPS structure and interaction with the Wzz proteins (Islam and Lam 2014) or differences in SDS‐PAGE migration as a function of the overall saccharide charge, as has been noted for other *Pseudomonas* OPS (King *et al*. 2008).

The genetic approach described herein enables product uniformity and enhanced yield of polysaccharides of a defined size. Should these polysaccharides present the optimum size for conjugation and immunity against the targeted pathogen, this would obviate the need for additional sizing steps that can introduce extraneous costs to the production process. Conceivably, it could be used to generate improved OPS haptens for several important GNB pathogens that use the Wzx‐/Wzy‐dependant pathway (e.g. *Salmonella*,* Shigella*,* Pseudomonas, Yersinia, Campylobacter, Acinetobacter, Francisella, Burkholderia, Escherichia coli*) and is an important advance towards enabling efficient use of OPS molecules as haptens by providing an interesting alternative strategy to facilitate carbohydrate vaccine development. Our team has already designed a highly immunogenic and protective *S*. Typhimurium glycoconjugate vaccine using *wzzB*‐controlled OPS antigen haptens, proof that this strategy is effective (Baliban *et al*. 2017). *Salmonella* Typhimurium overexpressing *wzzB* produced a single high molecular weight species of O‐polysaccharide as measured by HPLC‐SEC. In contrast, wild‐type *Salmonella* Typhimurium produced a bimodal size distribution. COPS was purified from the *wzzB* overexpresser using Tangential Flow Filtration (TFF) but no additional sizing steps thereby making purification simple and economical. In terms of immunogenicity, we previously observed that short‐chain OPS expressed by *S*. Typhimurium failed to induce significant antipolysaccharide antibody titres in the context of end‐linkage to a protein carrier (unpublished results), whereas the long‐chain form isolated from a *S*. Typhimurium strain overexpressing *wzzB* was highly immunogenic when linked with the identical chemistry. Others have also found that the long chain form of *S*. Typhimurium OPS is optimally immunogenic, however, the very long chain form was a poor vaccine hapten when end‐linked (Rondini *et al*. 2015). It has also been previously shown that the short‐chain COPS from *S*. *dysenteriae* was more immunogenic than the long form when linked through the reducing end to a protein carrier (Robbins *et al*. 2009). One proposed mechanism accounting for this latter observation was that the higher polysaccharide substitution ratio on the protein permitted by conjugation of shorter chain oligosaccharides enabled increased molar ratios of nonreducing end units, that are the immunodominant epitopes for this polysaccharide (Pozsgay *et al*. 2007). By comparison, for polysaccharides for which the central repeat units are immunodominant (e.g. *S*. Typhimurium OPS), the long‐chain polysaccharide form constitutes the desired hapten.

Various models attempt to explain the way *wzz* regulates OPS chain length (Islam and Lam 2014). It is likely that deeper understanding of that pathway could lead to a tunable method for controlling the synthesis of saccharides of an optimum size through overexpression of different *wzz* genes. Deleting the chromosomal *wzz* genes in the desired GNB or overexpressing the homologous *wzz* gene of a given GNB could also be explored to optimize the strategy described herein and maybe obtain even higher OPS yields of a desired size. The findings described in this study provide a proof of concept for applications whereby the desired saccharide population is not efficiently produced (e.g. glycoconjugate vaccine production) and work performed in our laboratory demonstrates that this strategy can be effectively used to improve glycoconjugate vaccine production.

## Conflicts of Interest

Drs. Tennant, Simon and Levine have a patent pending that includes the use of *wzz* to improve polysaccharide purification for conjugate vaccines: “Compositions and Methods for Producing Bacterial Conjugate Vaccines, PCT Patent Application Serial Number PCT/US2016/027325.” The authors declare no other conflicts of interest.
